# Laboratory evaluations of the 3-month efficacy of oral lotilaner (Credelio™) against experimental infestations of dogs with the Australian paralysis tick, *Ixodes holocyclus*

**DOI:** 10.1186/s13071-018-3061-8

**Published:** 2018-08-29

**Authors:** Kim Baker, Claudia Ellenberger, Martin Murphy, Daniela Cavalleri, Wolfgang Seewald, Jason Drake, Steve Nanchen, Kristina Hacket

**Affiliations:** 1 Elanco Animal Health, Yarrandoo Research and Development Centre, 245 Western Road, Kemps Creek, NSW 2178 Australia; 2 Elanco Animal Health, Mattenstrasse 24a, CH-4058 Basel, Switzerland; 3 Elanco Animal Health, 2500 Innovation Way, Greenfield, IN 46140 USA

**Keywords:** Credelio, Dog, *Ixodes holocyclus*, Lotilaner, Paralysis tick

## Abstract

**Background:**

From three days following host attachment, the Australian paralysis tick, *Ixodes holocyclus*, secretes a neurotoxin that annually causes paralysis in approximately 10,000 domestic pets. Lotilaner, a novel isoxazoline formulated in a chewable flavoured tablet (Credelio^TM^), produces rapid onset of acaricidal activity in dogs, with an efficacy duration of at least one month. Two studies were performed to determine the efficacy of lotilaner against *I. holocyclus* infestations over 3 months.

**Methods:**

Both studies included 16 dogs, ranked according to *I. holocyclus* counts on Day -5 (from infestations on Day -8) and blocked into pairs. One dog in each pair was randomized to be a sham-treated control, the other to receive lotilaner at a minimum dose rate of 20 mg/kg on Day 0. Dogs were dosed in a fed state. Infestations were performed in both studies on Days -8 (to determine the tick carrying capacity of each dog) -1, 28, 56, 70, 77 and 84, and additionally in Study 1 on Day 91, in Study 2 on Days 14 and 42. In Study 1, ticks were counted and assessed as alive or dead at 24, 48 and 72 h post-initial infestation and post-subsequent re-infestations. In study 2, ticks were counted at 24, 48 and 72 h post-dosing or post-re-infestation. Efficacy was determined by the percent reduction in live attached tick counts in the lotilaner group compared to control.

**Results:**

Within 48 h post-treatment in Study 1 and within 72 h post-treatment in Study 2 all lotilaner-group dogs were free of live ticks. By 72 h post-infestation, efficacy in Study 1 remained at 100% through Day 87, except on Day 31 when a single tick was found on one dog, and through Day 59 in Study 2. Efficacy exceeded 95% through the final assessment in each study (Days 94 and 87 in Studies 1 and 2, respectively).

**Conclusion:**

These results demonstrate that lotilaner quickly kills existing *I. holocyclus* infestations. By providing 95.3–100.0% protection through at least 87 days post-treatment, lotilaner can be a valuable tool in reducing the risk of tick paralysis in dogs.

## Background

The Australian paralysis tick *Ixodes holocyclus* is prevalent along the Australian eastern sea-board from Bairnsdale in south-eastern Victoria through New South Wales, north of Cairns as far as Shipton’s Flat in northern Queensland [1–3]. In addition to dogs, this tick has been known to cause paralysis in humans, cats, sheep, cattle, goats, pigs and horses, and is estimated to cause paralysis in 10,000 domestic pets annually [4]. In infested susceptible hosts, the appearance of clinical signs coincides with the rapid expansion of the tick’s salivary glands that produce a neurotoxin (holocyclotoxin) from three to five days post-attachment [5]. The result in affected hosts is a rapidly ascending flaccid paralysis which may culminate in death from respiratory failure. Although toy breeds and dogs less than six months are reported to be at greatest risk of tick-induced paralysis, infestation with a single tick may be fatal for a large dog or a sheep [6–8]. Detection of a single infesting *I. holocyclus* can be difficult, and attachment may occur in obscure sites such as between the digits, inside the anus or to the hard palate, sites where topically acting products may not readily achieve acaricidal concentrations. Systemically acting acaricides can therefore be valuable in preventing tick-induced paralysis.

In addition to paralysis, ticks in Australia can also vector a number of infectious diseases including *Rickettsia australis* which causes Queensland tick typhus and *Coxiella burnetti* which causes Q fever [1].

The potential for orally-administered isoxazolines, a family of novel compounds, to provide activity against insects and acari infestations for at least one month following oral administration sparked interest in molecular modifications that would enhance flea and tick control measures for dogs. A systematic evaluation utilizing rodent and *in vitro* studies screened a library of over 500 structures for efficacy and safety and led to the identification of lotilaner*.* Subsequent laboratory and field studies in dogs demonstrated that as a flavoured chewable tablet lotilaner (Credelio^TM^) is rapidly absorbed, is safe and provides efficacy against a number of tick species (*Ixodes scapularis*, *I. hexagonus*, *Haemaphysalis longicornis*, *Rhipicephalus sanguineus*, *Dermacentor reticulatus*, *D. variabilis*, *Amblyomma americanum* and *A. cajennense*) [9–15]. Lotilaner also has a rapid onset of activity against fleas and against the tick *Ixodes ricinus* from within four hours post-treatment [9–15]. In the investigations reported here, two studies were undertaken to determine whether the promising efficacy shown by lotilaner against tick infestations in dogs would be relevant to *I. holocyclus*.

## Methods

Both studies were designed and completed generally in accordance with the World Association for the Advancement of Veterinary Parasitology (WAAVP) guidelines for evaluating the efficacy of parasiticides for the treatment, prevention and control of flea and tick infestation on dogs and cats and the Australian Pesticides and Veterinary Medicines Authority (APVMA) Preamble for the WAAVP guideline for fleas and ticks on dogs and cats [16, 17]. All studies followed the principles of Good Clinical Practices as described in VICH guideline GL9, Good Clinical Practice (June 2000) [VICH] [18]. All personnel involved in completing tick infestations and assessments were blinded to treatment. Personnel who prepared and administered treatments and who performed post-dosing health checks did not participate in other study activities. The exception to this was on one occasion in Study 2 in which a dog required a clinical observation/examination for a non-treatment related adverse event.

### Animals and housing

For each study 20 dogs were acclimatized prior to selection for the study. From the 20 dogs, the 16 meeting the inclusion/exclusion criteria and with the highest pre-study tick counts were selected. In Study 1, the selected dogs were not hyper-immunised to the tick toxin and comprised an equal balance of sex and were a mix of cross- and pure-bred Huntaways, Beagles and Labradors. In Study 2 there were 10 males and 6 females that were hyper-immunised to the tick toxin and were a mix of pure-bred Foxhounds and cross-bred Foxhound/Bloodhound. Dogs in Study 1 were 2–9.5 years of age and weighed 29.1–47.2 kg pre-treatment. Dogs in Study 2 were 1.75–5.4 years of age and weighed 11.4–22.0 kg pre-treatment. Inclusion criteria were that all dogs were healthy, neither pregnant nor lactating, had to have retained at least 50% of a burden from a tick challenge completed on Day -8, and were free of the effects of any previous acaricidal treatment (evidenced by retention of a pre-study challenge and medical records). All dogs were identified with a subcutaneous microchip identification number. In both studies, dogs were individually housed on days when ticks were attached. On other days the dogs were housed in suitable pairs within each treatment group, except for isolated occasions where dogs could not be suitably paired. In study 1, dogs had access to outside runs for exercise (dogs from same treatment groups together) in the time between first and second tick attachment (day 7 to day 24). In Study 2, on non-infestation days dogs were exercised in outdoor grassed runs for at least one hour each day, and dogs from the same group were exercised together, separate from the other group. Dogs were fed a commercially available high quality complete canine diet and water was provided *ad libitum* from automatic drinkers or self-filling water bowls.

### Tick infestations and counts

**Fig. 1:**
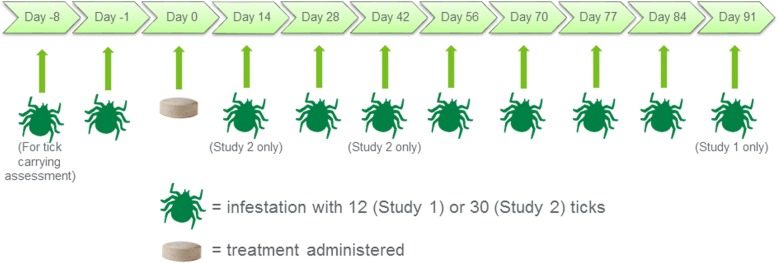
Timing of infestations and treatment during Study 1 and Study 2

The *I. holocyclus* ticks used in each study were sourced from wild strains collected from the Northern Rivers district of NSW and into southern Queensland, six to nine months prior to use. Initial infestations were performed in April and continued into July of the same year for each study. Infestation involved parting the hair coat and placing unfed adult female ticks on the skin, one at a time. Attachment was confirmed by gently tugging or by brushing over the tick. In Study 1 dogs were lightly sedated with 0.02–0.1 mg/kg acepromazine maleate for infestations which were with 12 ticks, evenly balanced between the left and right side of the head and/or neck (one treated dog had 10 ticks placed on Day 28 and another treated dog had 11 placed on Day 84). Ticks that could not be attached in approximately 10 min were replaced by another tick. In Study 2, in non-sedated dogs, anatomic sites for the 30 female ticks used in each infestation were the ears, forehead, lips, under the eyes, between the shoulders, on each shoulder, and on the mid-back and tail base. Infestations were completed in both studies on Days -8 (for ranking and demonstrating tick viability), -1, 28, 56, 70, 77 and 84 and, additionally, in Study 1 on Day 91, and in Study 2 on Days 14 and 42 (Fig. 1). The decision to use different numbers of ticks in each study was determined by the experimental infection models which were developed in each facility. The numbers of ticks infested were in compliance with the WAAVP 2013 guidelines. Tick counts involved careful examination of infestation sites and, if ticks were missing from the infestation sites, a whole-body search was conducted to ensure that all ticks were observed. Counts of *in situ* ticks were made 24 h post-treatment (the day following treatment) in both studies and 48 h post-treatment in Study 2, and then at 24 and 48 h following each post-treatment infestation. Ticks were counted and removed on Days -5 in both studies, 48 h post-treatment in Study 1 and 72 h post-treatment in Study 2, and at 72 h following each post-treatment infestation. Ticks were considered alive if they demonstrated active leg movement, dead ticks showed no leg movement, did not react when stimulated, and may have appeared crenated or desiccated.

### Randomization and treatments

Using tick counts made on Day -5, dogs that met all inclusion and no exclusion criteria were ranked, in descending order, and within sex in Study 1. The 16 dogs with the highest tick counts were then included in the randomization process. The two dogs with the highest tick counts were allocated to form a block, the next two a second block, and so on to form 8 blocks. Dogs within each block were assigned using random order numbers to either treatment with lotilaner or to be a sham-treated control. All dogs had consumed at least a partial daily ration within approximately 30 min before dosing. Lotilaner’s bioavailability is increased when administered in the fed state [9].

Lotilaner was administered orally (whole tablets) at as close as possible to the targeted minimum dose rate of 20 mg/kg. Each dog was dosed based on individual body weight determined to an accuracy of 0.1 kg (Day -5: Study 1 and Day 0: Study 2). Following tablet administration 5 to 10 ml of water was administered orally *via* a syringe to help with swallowing. Control group dogs were sham-dosed on Day 0 using a process that matched the handling of the lotilaner-treated dogs, including removing the dog from its accommodation, opening the dog’s mouth and administering 5 to 10 ml of water.

### Assessment of efficacy

Efficacy was determined by the percent reduction in live attached tick counts in the lotilaner-treated group compared to the untreated control group. Arithmetic and geometric mean group efficacies were calculated as follows using the equation:

$$ \mathrm{Efficacy}\ \left(\%\right)=100\times \left(\mathrm{Mc}-\mathrm{Mt}\right)/\mathrm{Mc} $$

where Mc is the arithmetic mean number of live ticks on dogs in the untreated control group and Mt is the arithmetic mean number of live ticks on dogs in the treated group. Geometric means were also calculated using logarithm transformed counts (count + 1) with one (1) subsequently subtracted from the result.

For the first study, tick counts for each day were log-transformed and analyzed with an ANOVA and treatment as the only model parameter. For the second study, tick counts were analyzed with a generalized linear model with binomial distribution and log link, and the following model parameters: treatment, day, treatment/day interaction. All hypotheses were tested at a two-sided 0.05 level of significance. Lotilaner was considered effective if there was a minimum of 50% tick retention rate in at least six control dogs and at least a 95% reduction in mean live tick counts in the treated group, compared to the control group, with a significant between-group difference (*P* < 0.05) in live tick counts [16]. For Study 1 the tick attachment in the untreated dogs ranged from 87.5–100% from day 1 to 94 and in Study 2 it ranged from 74.2–91.3% from day 1 to 87. All calculations were carried out using the software SAS®, Version 9.2.2.

## Results and discussion

In both studies infestations in the control dogs were adequate at all time-points. In Study 1 the mean infestation rate in the control dogs ranged from 87.5 to 100% after each challenge. In Study 2, the mean infestation rate in the control group ranged from 74.2 to 91.3% of the challenge burden.

**Fig. 2:**
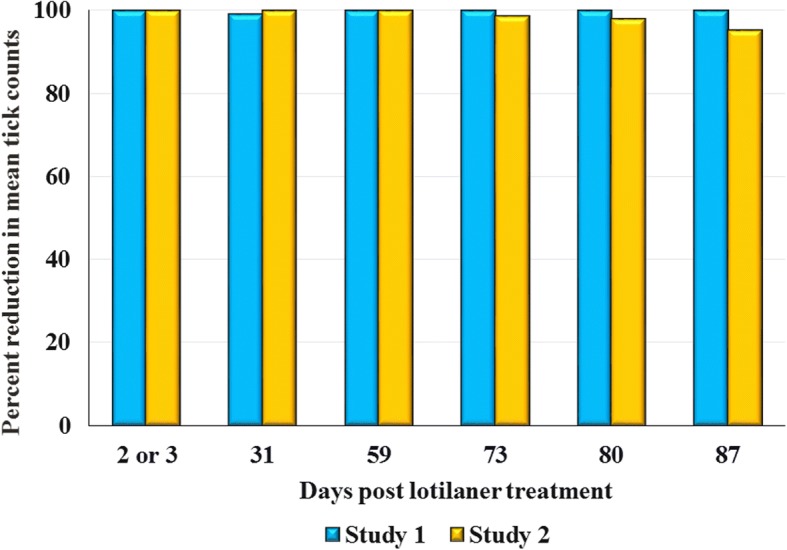
Percent reduction in geometric mean *Ixodes holocyclus* counts of lotilaner-treated dogs compared to untreated control group dogs 72 h following each infestation post-treatment (showing assessments days common to Study 1 and Study 2)

Across the two studies, within 48 h post-treatment all lotilaner-treated dogs were free of live ticks, demonstrating efficacy of 100% or close to 100%; in Study 1, a single live tick was found on one lotilaner-treated dog 72 h following the infestation on Day 28. For other assessments completed at 72 h after post-treatment infestations efficacy remained at 100% through Day 59 in Study 2 and through Day 87 in Study 1 (Tables 1, 2; Fig. 2).

**Table 1:** Study 1 counts in untreated control and lotilaner-treated dogs when live ticks were removed 48 h post-treatment and 72 h following each post-treatment infestation

Day of study	Arithmetic mean ± SD		Geometric mean		*t*-value	*P*-value	Efficacy (*%*)^a^
	Control	Lotilaner	Control	Lotilaner			
2	11.4 ± 1.1	0.0 ± 0.0	11.3	0	*t*_(14)_ = 77.2	<0.0001	100
31	11.6 ± 0.7	0.1 ± 0.4	11.6	0.1	*t*_(14)_ = 27.4	<0.0001	99.2
59	11.5 ± 1.1	0.0 ± 0.0	11.5	0	*t*_(14)_ = 76.7	<0.0001	100
73	10.8 ± 1.3	0.0 ± 0.0	10.7	0	*t*_(14)_ = 62.1	<0.0001	100
80	10.5 ± 0.9	0.0 ± 0.0	10.5	0	*t*_(14)_ = 85.2	<0.0001	100
87	10.9 ± 1.0	0.0 ± 0.0	10.8	0	*t*_(14)_ = 81.0	<0.0001	100
94	11.0 ± 0.9	0.4 ± 0.5	11.0	0.3	*t*_(14)_ = 17.1	<0.0001	97.3

^a^Efficacy based on geometric means

*Abbreviation*: SD, standard deviation

**Table 2:** Study 2 tick counts in untreated control and lotilaner-treated dogs when live ticks were removed at 72 h post-treatment and at 72 h following each post-treatment infestation

Day of study	Arithmetic mean ± SD		Geometric mean		*t*-value	*P*-value	Efficacy (*%*)^a^
	Control	Lotilaner	Control	Lotilaner			
3	22.3 ± 3.5	0.0 ± 0.0	22.0	0	†	†	100
17	24.1 ± 1.3	0.0 ± 0.0	24.1	0	†	†	100
31	25.9 ± 2.0	0.0 ± 0.0	25.8	0	†	†	100
45	23.4 ± 4.3	0.0 ± 0.0	23.0	0	†	†	100
59	24.6 ± 2.3	0.0 ± 0.0	24.5	0	†	†	100
73	23.6 ± 2.7	0.8 ± 2.1	23.5	0.3	*t*_(266)_=8.5	<0.0001	98.8
80	26.1 ± 1.6	2.9 ± 8.1	26.1	0.5	*t*_(266)_=11.0	<0.0001	98.1
87	26.3 ± 3.9	4.1 ± 8.4	26.0	1.2	*t*_(266)_=11.3	<0.0001	95.3

^a^Efficacy based on geometric means

*Abbreviation*: SD, standard deviation

†For efficacy values of 100%, the statistical method does not produce a t- or *P*-value

The use of hyper-immunized dogs in experimental infestations studies with *I. holocyclus* is recommended by the WAAVP 2013 guidelines. This recommendation serves to provide additional protection to the dogs should a tick stay on the animal > 96 h by which time toxin will have been injected and the clinical signs of toxicosis will start to be visible. The use of naïve dogs in Study 1 demonstrated that with the appropriate animal welfare safeguards in place, it is possible to conduct such experimental infection studies in non-hyper-immunized dogs.

The dose rates of lotilaner administered to study dogs ranged from 20.2–24.7 mg/kg. Vomitus was found in the pen of one dog in Study 2 in the lotilaner group at six and eight hours post-treatment and again at the 72 h post-treatment examination. The possibility that this was treatment related could not be excluded. In Study 2, 48 h after lotilaner treatment a dog was observed with pyrexia (39.8 °C) and bilateral conjunctivitis, and developed clinical signs consistent with pneumonia. The cause of the respiratory signs that developed are unknown but may have been a consequence of aspiration of water administered as part of the dosing process as required by the study protocol. The dog responded to treatment with amoxicillin/clavulanic acid and meloxicam, and on Day 35 underwent surgery to rectify entropion, which was identified as the cause of the bilateral conjunctivitis. The time taken to kill ticks and duration of lotilaner protection for this dog declined more rapidly than for the other treated dogs, and this dog was the only dog with live ticks identified at the time of tick removal on Days 73 and 80.

The findings of lotilaner efficacy against *I. holocyclus* in this study parallel those of a report of the efficacy of sarolaner and afoxolaner in eliminating existing burdens and post-treatment infestations through 35 days after treatment [19]. Extending well beyond the findings of that study, we have demonstrated the potency of lotilaner in providing a high level of efficacy through at least 12 weeks after treatment against *I. holocyclus* when evaluated at 72 h post-treatment or re-infestation. The efficacy of lotilaner is in line with a report of the 72 hour efficacy of fluralaner against *I. holocyclus* being sustained through 115 days post-treatment [20]. This extended activity against *I. holocyclus* is beyond the demonstrated efficacy duration of lotilaner against other tick species like *R. sanguineus*, *D. variabilis*, *D. reticulatus* and *A. americanum*, suggesting that the Australian paralysis tick is more susceptible to lotilaner than those other species [12, 13]. Nonetheless, no product can be expected to be fully effective on 100% of occasions. Regardless of the protective strategy for *I. holocyclus,* vigilance by owners and veterinarians remains important in the prevention of tick-induced paralysis.

Regular and reliable treatment of dogs with ectoparasiticides can maximize protection against arthropod challenge, and to that end, lotilaner has been shown to provide at least 30 days activity against fleas and a wide range of tick species and, formulated as Credelio™, is indicated for monthly use in dogs. This is particularly relevant to the risk of infestation with *I. holocyclus* as compliance failure has been indicted as an important factor contributing to cases of tick paralysis in dogs [4]. Owners should therefore be directed to be rigid in administering scheduled treatments. However, adherence to this direction may not always be complete. For such lapses, this study demonstrates that the high and sustained efficacy of lotilaner against *I. holocyclus* allows a period of “forgiveness” should there be any delay of dosing beyond the monthly schedule.

## Conclusion

These studies demonstrate that lotilaner given orally at a minimum dose rate of 20 mg/kg is well tolerated and eliminated existing *I. holocyclus* tick infestations within 48 hours of treatment. By providing 95.3–100.0% protection through at least twelve weeks post-treatment lotilaner can be a valuable tool in reducing the risk of tick paralysis in dogs.

## Abbreviations

Mc: mean number of live ticks on dogs in the untreated control group
Mt: mean number of live ticks on dogs in the treated group
SD: standard deviation
VICH: International Cooperation on Harmonization of Technical Requirements for Registration of Veterinary Medicinal Products
WAAVP: World Association for the Advancement of Veterinary Parasitology
